# Altered Spontaneous Brain Activity in Cirrhotic Patients with Minimal Hepatic Encephalopathy: A Meta-Analysis of Resting-State Functional Imaging

**DOI:** 10.3390/brainsci13060960

**Published:** 2023-06-16

**Authors:** Bin Qin, Shuolin Liang, Shiting Tang, Huo Liang, Yunli Zhang, Zhijian Liang

**Affiliations:** Department of Neurology, The First Affiliated Hospital of Guangxi Medical University, Nanning 530021, China; bin13457@163.com (B.Q.); nnlsl093@163.com (S.L.); tst-tingting@163.com (S.T.); gxlh68@outlook.com (H.L.); yunliyou@163.com (Y.Z.)

**Keywords:** minimal hepatic encephalopathy, functional neuroimaging, magnetic resonance imaging, meta-analysis

## Abstract

(1) Background: Minimal hepatic encephalopathy (MHE) is an important complication of decompensated cirrhosis. Previous studies have demonstrated spontaneous brain activity alterations in cirrhotic patients with MHE. However, the reported results are inconsistent, which has limited our understanding of the potential neural mechanisms. Thus, we conducted a quantitative meta-analysis of resting-state functional imaging studies to identify the regional activity alterations consistently involved in MHE. (2) Methods: We searched six databases to include resting-state functional imaging studies and compared spontaneous brain activity patterns between MHE patients and healthy controls (HCs), and between cirrhotic patients without minimal hepatic encephalopathy (NMHE) and HCs. Then, a separate whole-brain voxel-wise meta-analysis between MHE or NMHE patients and HCs was conducted using seed-based d mapping with permutation of subject images. We further conducted the conjunction analysis to assess the distinct regional activity alterations between MHE and NMHE patients as compared to HCs. (3) Results: Thirteen studies with twenty datasets were included in this meta-analysis. Compared with HCs, MHE patients showed decreased spontaneous brain activity in the left superior frontal gyrus, left median cingulate/paracingulate gyri, and right precuneus. Compared with NMHE patients, MHE patients indicated decreased spontaneous brain activity in the left superior frontal gyrus, left median cingulate/paracingulate gyri, and right precuneus. (4) Conclusions: MHE is associated with spontaneous brain activity alterations involving the left superior frontal gyrus and median cingulate/paracingulate gyri, which may implicate primarily in spatial working memory and emotional disorders. These findings may contribute to a better understanding of the potential neural mechanisms, and guide further research.

## 1. Introduction

Hepatic encephalopathy (HE) is a major complication of decompensated liver disease and is highly prevalent in cirrhosis. The earliest and mildest form of HE is known as minimal hepatic encephalopathy (MHE), which was previously referred to as subclinical HE. It is characterized by slow alterations in psychomotor and neurocognitive functioning, such as a decline in working memory, visuospatial disability, emotional and attention deficits, and it is difficult to detect in a conventional physical examination [1,2]. Consequently, MHE is the most under-recognized and under-diagnosed form of HE [3]. It is widely recognized that more than 30% of patients with cirrhosis suffer from MHE, and as many as 80% of patients with chronic liver disease [3,4]. Currently, there is no gold standard for diagnosing MHE; however, several validated testing modalities have been developed to identify this neurocognitive complication [3]. This is clumsy and time-consuming; thus, these tests are not routinely administered to patients by most clinicians [5]. Despite the lack of obvious neuropsychiatric syndrome, MHE significantly affects the health-related quality and daily functioning of patients. Moreover, MHE predicts a higher risk of progressing to overt HE, which is associated with a poor prognosis and a higher risk of overall mortality rates [5,6,7]. Thus, the early identification of, and prompt intervention for, MHE are significant clinical importance, as they can potentially reduce the socioeconomic burden associated with the disease and ameliorate the quality of life [2,3].

However, the pathophysiology of MHE is extremely complicated and remains elusive. Recently, resting-state functional magnetic resonance imaging (rs-fMRI), a powerful and noninvasive tool for exploring intrinsic spontaneous brain activity, has been widely used to investigate and uncover the potential neural mechanisms of various diseases [8]. Many algorithms have been used to analyze rs-fMRI data, such as the amplitude of low-frequency fluctuations (ALFF), fractional ALFF (fALFF), and regional homogeneity (ReHo); these algorithms can provide us with information about regional spontaneous brain activity [9].

Over the past decade, a number of rs-fMRI studies investigating spontaneous brain activity alterations in cirrhotic patients with MHE have been increasingly published [10,11,12,13,14]. However, there has been some variation in the results of these studies, and some even showed the opposite results. On one hand, different directions of spontaneous brain activity of the same brain regions were reported between studies. For instance, some studies showed decreased ALFF in the inferior frontal gyrus [12,14], while another study detected increased ALFF in the same region [10]. On the other hand, different brain regions were reported in different studies, such as different spontaneous brain activities in the cuneus and supplementary motor area [11], default-mode network (DMN) [12], and visual network and somatomotor network [13]. Additionally, in different studies, decreased ALFF was also observed along with increased ReHo in the superior frontal gyrus [12,13,14]. Multiple factors contribute to these inconsistent results, such as a small sample size with heterogeneous patient etiology, leading to a low level of statistical power and a high probability of false positives. This inconsistency impedes the comprehension of the potential neural mechanisms of MHE. Further studies are needed to advance this field. Thus, combining existing relevant rs-fMRI studies and performing a comprehensive meta-analysis is necessary to gain a better understanding of the potential neural mechanisms of MHE.

Quantitative neuroimaging meta-analysis can be a powerful approach for pooling individual original studies to distinguish spurious results and produce reliable results, among which size-signed differential mapping and activation likelihood estimation are the most commonly used methods. Seed-based d mapping with permutation of subject images (SDM-PSI), a recently developed size-signed differential mapping approach, is an analytical technique used in meta-analyses of neuroimaging studies that examine differences in brain activity or structure [15]. In the new SDM-PSI method, subject images are imputed to allow for subject-based permutation tests, a less biased estimate of the population effect size, and multiple imputations of study images in order to minimize the biases associated with single imputation. Heretofore, neuroimaging meta-analyses widely use SDM-PSI, which is considered an effective tool. Moreover, the algorithms, such as the ALFF, fALFF and ReHo, can provide us with information about regional spontaneous brain activity, and this makes it possible to study the changes in spontaneous brain activity by merging them. Accordingly, a comprehensive synthesis and meta-analysis of resting-state functional imaging studies was conducted to identify the regional activity alterations consistently involved in MHE in this study. We aimed to (I) identify regional spontaneous brain activity alterations in patients with MHE compared with healthy controls (HCs) and cirrhotic patients without MHE (NMHE), and (II) investigate the potential correlations between clinical variables and regional spontaneous brain activity alterations.

## 2. Materials and Methods

This meta-analysis followed the preferred reporting items for systematic reviews and meta-analyses guidelines [16].

### 2.1. Data Sources and Search Strategies

The following electronic databases were searched to identify all relevant studies: PubMed, Cochrane Library, Web of Science, Embase, China Science and Technology Journal Database, and China National Knowledge Infrastructure core journals. The last search was conducted in December 2022 using the following terms: (cirrhosis OR cirrhotic OR “liver cirrhosis” OR “hepatic fibrosis” OR “hepatic encephalopathy” OR HE OR “minimal hepatic encephalopathy” OR MHE) AND (“functional magnetic resonance imaging” OR “functional MRI” OR fMRI OR “amplitude of low-frequency fluctuation” OR ALFF OR fALFF OR “regional homogeneity” OR ReHo) (Table S1). This search strategy was revised to be appropriate for the Chinese electronic databases (China Science and Technology Journal Database, and China National Knowledge Infrastructure). Furthermore, we manually reviewed the reference lists of the included studies, reviews, and meta-analyses.

### 2.2. Study Selection

The study inclusion criteria were as follows: (I) an original study comparing MHE or NMHE patients against HCs on regional spontaneous brain activity; (II) use of ALFF, fALFF, or ReHo methods to detect brain activity; (III) the neuroimaging results were reported in whole-brain three-dimensional coordinates (x, y, z) in standard stereotactic space (Talairach or Montreal Neurological Institute); and (IV) thresholds for significance were corrected for multiple comparisons. The neuropsychologic tests have been used to identify patients with MHE as reported in previous studies [3,5]. In addition, according to most included studies in this meta-analysis, the number connection test-A (NCT-A) and digit-symbol test (DST) were the common neuropsychologic tests. Consequently, the neuropsychiatric tests, primarily including NCT-A and DST, were used to define MHE in this meta-analysis. Studies were excluded based on the following criteria: (I) it did not provide coordinates despite contact with the author; (II) studies that were duplicated or non-original; and (III) it involved less than 10 participants per group. The titles and abstracts of the search citations were independently screened by two experienced investigators (B.Q. and S.L.), full articles were reviewed, and the eligibility of the candidate studies was determined. Any discrepancies were resolved through discussions with other investigators (Z.L. and S.T.).

### 2.3. Data Extraction and Quality Assessment

Two investigators (B.Q. and S.L.) independently extracted the summary data that were utilized in the analysis. The peak coordinates and effect sizes of significant alterations in both directions (i.e., patients > HCs and patients < HCs) of each study were independently extracted in accordance with SDM-PSI requirements. A 10-point checklist was used to evaluate the quality of each eligible study, based on the criteria used in previous neuroimaging meta-analyses for evaluating study quality [17,18]. (Table S2). Two independent investigators evaluated each eligible study (B.Q. and S.L.), and a third investigator was consulted to resolve disagreements (Z.L.).

### 2.4. Data Synthesis and Analysis

#### 2.4.1. Voxel-Based Meta-Analysis

A separate meta-analysis of regional differences in spontaneous brain activity between MHE or NMHE patients and HCs, respectively, was conducted using SDM-PSI (version 6.22, https://www.sdmproject.com/ [accessed on 5 February 2023]) [15]. It is a recently developed, size-signed differential mapping, quantitative, coordinate-based meta-analytic technique that can reconstruct effect-size maps by combining whole-brain t-maps with peak coordinates of statistical significance. The SDM-PSI method has previously been described in detail [19]. To assign higher effect sizes to voxels that tend to be more correlated with peaks, the wide full-width at half-maximum was set to 20 mm half-width [20]. A threshold-free cluster enhancement (TFCE)-based family-wise error rate-corrected threshold *p* < 0.05, with a voxel extent ≥10 was primarily used throughout the analyses [15], and the results were shown in the Montreal Neurological Institute coordinates. The final outcome (the SDM map) was visualized using MRIcron software (http://www.mccauslandcenter.sc.edu/mricro/mricron/ [accessed on 5 February 2023]) on a brain template generated by the International Consortium for Brain Mapping.

The interstudy heterogeneity of individual clusters was quantified by calculating the inconsistency index (I^2^) [19], in which a value of 0% to 25% indicated mild heterogeneity and >50% indicated substantial heterogeneity [21]. Then, the asymmetry of funnel plots was tested using the Egger test to detect publication bias for each significant cluster, in which any result showing *p* < 0.05 was regarded as having significant publication bias [22].

#### 2.4.2. Conjunction Analysis between Meta-Analysis Groups

Following the separate analyses between MHE or NMHE patients and HCs, we further conducted comparative analyses to assess whether there were any distinct spontaneous brain activity alterations across the two groups by comparing patients with MHE and NMHE. Conjunction analyses can be conducted with SDM-PSI to compare the outcomes of different meta-analytic groups (for instance, MHE versus NMHE patients as compared to HCs). In particular, for meta-analytic group comparisons, a linear modal tool was used to examine whether computed effect sizes differed significantly between the groups [23].

#### 2.4.3. Subgroup Meta-Analysis and Meta-Regression Analysis

To control for the effects of latent factors on the main results, we conducted subgroup meta-analyses regarding the analysis method (ALFF/fALFF and ReHo algorithm) and scanner strength (3.0 T and 1.5 T). The same statistical thresholds were set as in the main analysis (TFCE-corrected *p* < 0.05, extent >10 voxels). Meta-regression analyses were conducted to evaluate the correlations of clinical variables and spontaneous brain activity alterations in patients with MHE and HCs. To minimize the detection of spurious relationships, a stringent uncorrected threshold of *p* < 0.0005 was adopted [23]. To ensure that the results were significant in the primary meta-analysis, we required the findings to be detected both in the slope and at one of the extremes of the regression.

## 3. Results

### 3.1. Included Studies and Sample Characteristics

**Figure 1:**
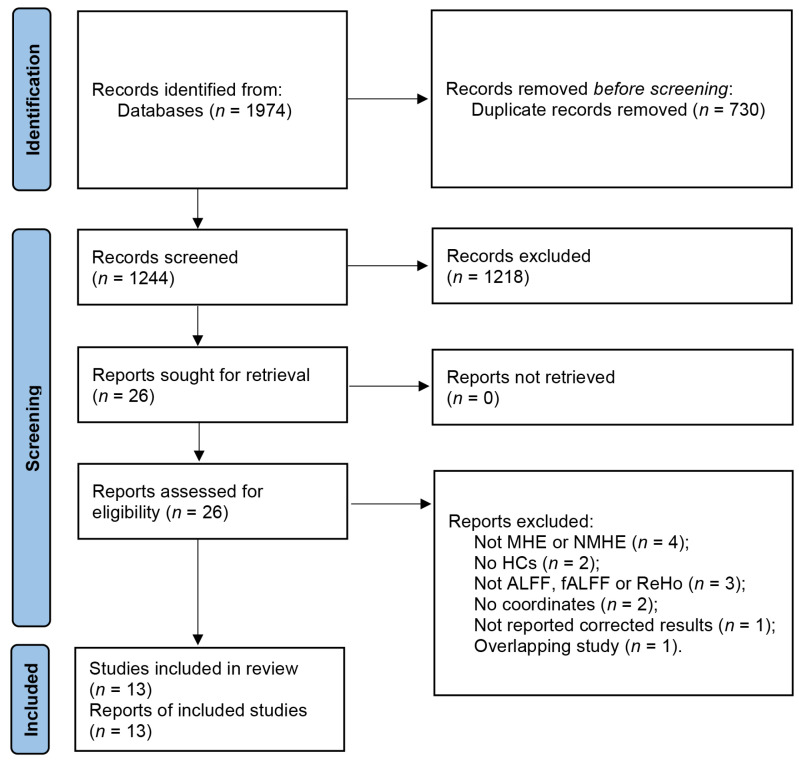
Flow diagram of studies included and excluded at each stage of identification and verification, following the preferred reporting items for systematic reviews and meta-analyses guidelines.

The search strategy resulted in the identification of 1974 relevant articles. After removing duplicates, a total of 1244 articles were screened, of which 13 studies with 20 datasets were deemed eligible for our meta-analysis. This included 13 datasets for the analysis of patients with MHE and 7 datasets for the analysis of patients with NMHE (Figure 1) [10,11,12,13,14,24,25,26,27,28,29,30,31]. Detailed demographic and clinical information about each study is provided in Table 1, as well as the summarized sample sizes (257 MHE patients versus 296 HCs, and 179 NMHE patients versus 219 HCs); the female ratio was 29.6% and 34.5% in MHE patients and HCs groups, respectively, and 29.1% and 35.2% in NMHE patients and HCs, respectively. Mean ages of MHE patients (51.85 ± 4.35 years) and HCs (50.90 ± 3.69 years) groups were matched (t = 0.578, *p* = 0.466, two-sample *t*-test), and NMHE patients (47.35 ± 2.95 years) and HCs (48.58 ± 3.80 years) were not significantly different (t = −0.629, *p* = 0.459, two-sample *t*-test). Mean education years of MHE patients (8.23 ± 1.36 years) and HCs (9.03 ± 1.33 years) were not significantly different (t = −1.264, *p* = 0.486, two-sample *t*-test), as well as NMHE patients (8.91 ± 1.90 years) and HCs (9.12 ± 1.67 years) were not significantly different (t = −0.202, *p* = 0.656, two-sample *t*-test). The quality assessment scores of the included studies are presented in Table S3, while a summary of the neuroimaging methodologies used is shown in Table S4. The range of quality scores was 9–10, with an average score of 9.6 points for the included studies.

### 3.2. Results of the Main Meta-Analysis

#### 3.2.1. MHE Patients versus HCs

**Figure 2:**
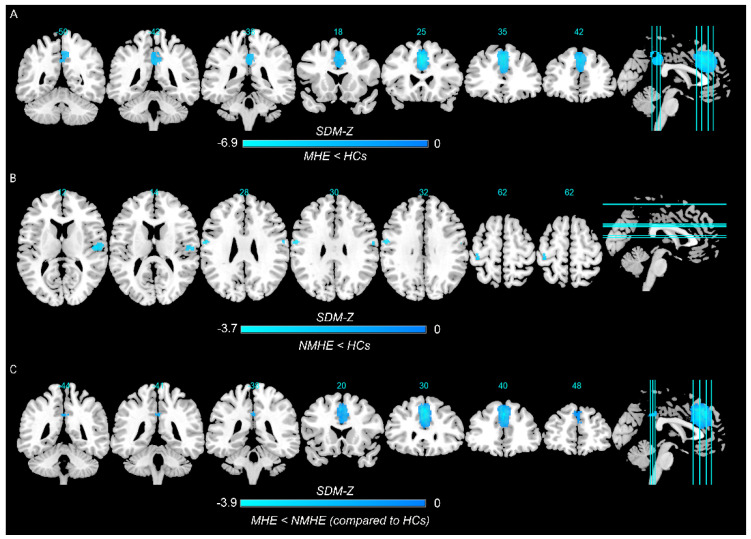
Regions showing spontaneous brain activity alterations in (**A**) minimal hepatic encephalopathy (MHE) patients compared with healthy controls (HCs), (**B**) cirrhotic patients without MHE (NMHE) compared with HCs, and (**C**) between MHE and NMHE patients as compared to HCs. *Note: Cold color: decreased; Warm color: increased; SDM−Z, seed−based d mapping z score*.

In the pooled meta-analysis, in contrast to HCs, MHE patients showed decreased spontaneous brain activity in the left superior frontal gyrus, left median cingulate/paracingulate gyri, and right precuneus, whereas there was no significant increase in regional spontaneous brain activity (Figure 2A and Table 2).

#### 3.2.2. NMHE Patients versus HCs

The pooled meta-analysis analyzed differences in regional spontaneous brain activity between NMHE patients and HCs, and there was no suprathreshold cluster between NMHE patients and HCs (seven datasets) when the threshold was set at TFCE-corrected *p* < 0.05 and extent >10 voxels. When applying a more liberal threshold (*p* < 0.005, uncorrected, with a cluster extent of at least 10 voxels), decreased spontaneous brain activity was observed in the right rolandic operculum, left precentral gyrus, bilateral postcentral gyrus, and right superior temporal gyrus (Figure 2B and Table 2).

#### 3.2.3. Conjunction Analysis between MHE and NMHE Patients as Compared to HCs

The conjunction analysis showed that MHE patients had decreased spontaneous brain activity in the left superior frontal gyrus, bilateral median cingulate/paracingulate gyri, and right precuneus than patients with NMHE (Figure 2C and Table 2). There was no significant increase in regional spontaneous brain activity in patients with MHE.

### 3.3. Subgroup Meta-Analysis and Meta-Regression Analysis

Regional spontaneous brain activity alterations in subgroup meta-analyses concerning the analysis approach (fALFF/ALFF and ReHo algorithm) remained consistent with those in the main analysis (Tables S5 and S6). The subgroup meta-analyses concerning scanner strength (3.0 and 1.5 T) were not considered, because most included studies reported scanner strength with 3.0 T, except only three studies with 1.5 T. For MHE patients, variables explored by regression are the mean age (available in all the studies), percentage of women (available in all the studies), years of education (nine studies with ten datasets), and the mean DST (eight studies with nine datasets), but the results showed these variables were not significantly associated with regional spontaneous brain activity alterations. Because there were fewer than nine datasets available for analysis, the following variables were not analyzed: mean NCT-A, Child–Pugh scale, and mean ammonia levels. In addition, meta-regression analysis was not conducted in NMHE patients, despite including only seven datasets.

### 3.4. Analyses of Heterogeneity and Publication Bias

The heterogeneity analysis results are shown in Table 2. No significant between-study heterogeneity was observed in any of the clusters mentioned above. According to the Egger test, publication bias was not significant in abnormal regions for MHE patients versus HCs, NMHE patients versus HCs, or between MHE and NMHE patients (Table 2).

## 4. Discussion

By conducting a quantitative meta-analysis utilizing the most recent size-signed differential mapping approach (SDM-PSI version 6.22) with a TFCE-corrected threshold, our results showed that spontaneous brain activity consistently decreased in the left superior frontal gyrus, left median cingulate/paracingulate gyri, and right precuneus in patients with MHE relative to HCs. Furthermore, the spontaneous brain activity of MHE patients was also decreased in the right precuneus, bilateral median cingulate and paracingulate gyri, and left superior frontal gyrus compared to NMHE patients.

At the superior part of the prefrontal cortex, a variety of cognitive and motor control tasks are performed by the left superior frontal gyrus in previous studies [32,33]. Specifically, the left superior frontal gyrus is involved in the execution of tasks within the domains of working memory and attention, as well as cognitive-related processing, and is a component of the DMN [33]. Patients with lesions in the left superior frontal gyrus displayed deficits in working memory, involving verbal, spatial, and face stimuli, which is the strongest evidence for its role in working memory [34]. In another study, electrocorticography and direct cortical stimulation were combined to assess three patients implanted with subdural electrodes; the results also indicated that the left superior frontal gyrus plays a functional role in working memory [35]. Furthermore, the previous study demonstrated that attention, higher executive functions, and memory deficits are the main clinical presentation components of MHE patients [36]. According to the findings of an ethology and fMRI study, MHE patients debilitate spatial working memory, and neural network impairments result in spatial working memory dysfunction [37]. Consequently, the present study found decreased spontaneous activity in the left superior frontal gyrus, which is involved in working memory domains in MHE patients relative to HCs or NMHE patients. This finding is consistent with the primary clinical manifestations of MHE patients, such as working memory deficits. The results of this study with the current comprehensive analysis strengthen the evidence that the left superior frontal gyrus may play a crucial role in the potential neural mechanisms of patients with MHE, and MHE patients with impairments of the left superior frontal gyrus may exhibit deficits in working memory. Despite our expectations, no correlation was found between the values in this area and neuropsychological tests. Regardless of the severity of symptoms, we speculated that decreased spontaneous activity might be a trait alteration associated with MHE patients. Additionally, the relatively small sample size might have contributed to the confounding factors. Furthermore, a previous meta-analysis of rs-fMRI studies reported no correlation between abnormal spontaneous brain activity and DST score in patients with liver cirrhosis, which is consistent with our findings [38].

This study also found that resting-state neural activity was decreased in the median cingulate/paracingulate gyri in patients with MHE. A previous meta-analysis (which only included six studies) on patients with liver cirrhosis has shown that MHE is associated with decreased brain activity in the bilateral cingulate gyri, suggesting a disruption in local brain activity fluctuations [38]. This finding is consistent with our study, which has documented altered resting-state neural activity in the cingulate/paracingulate gyri in patients with MHE. Compared with the previous study, our study covered the latest published studies (12 studies with 13 datasets), specifically for patients with MHE, and adopted TFCE-corrected threshold approaches, which were neither too conservative nor too liberal in the simulation work [39]. There has been a decrease in gray matter volume in the cingulate/paracingulate gyri in patients with MHE [40], which may explain our findings on a structural basis. It is known that negative emotions are associated with aberrant activity of the cingulate/paracingulate gyri in the limbic system, which is responsible for regulating emotional disorders [41]. It has also been observed that paracingulate activity is also associated with executive and higher-order processing tests, such as spatial working memory and planning [42,43,44]. Furthermore, in the mild expression of MHE patients, it is associated with impaired performance on psychometric tests, including those measuring working memory, psychomotor speed, and visuospatial abilities [45]. It is understood that HE is comprised of cognitive, affective/emotional, behavioral and bioregulatory deficits [46]. Another study also found that cirrhotic patients are more likely to experience depression [47]. Thus, dysfunction in the cingulate/paracingulate gyri may be associated with impairments in emotional regulation and working memory, corresponding with the clinical manifestations of MHE patients, which include cognitive and affective/emotional deficits.

Additionally, this study found that a small number of brain regions exhibited functional changes in patients with MHE, including decreased spontaneous activity in the right precuneus. In a previous study involving diffusion tensor imaging and fMRI, MHE patients have demonstrated increased mean diffusivity and decreased fractional anisotropy in the precuneus [48]. There is considerable interest in the precuneus because of the fact that it is buried in the posteromedial cortex of the parietal lobe, as well as its possible function in fundamental cognitive and highly integrated tasks [49]. Moreover, the study by Utevsky et al. indicated that the precuneus plays a critical role in DMN [50], and a number of cognitive and affective functions are carried out by the DMN [51]. In patients with MHE, focal damage was found in the precuneus and alterations to the microstructure of white matter correlated with cognitive dysfunction [52,53]. These findings are consistent with our study’s results, which suggests that changes in the right precuneus may be associated with various cognitive and affective dysfunctions in patients with MHE.

The discovery and measurement of MHE poses significant challenges in clinical practice [40]. Recently, artificial intelligence (AI) and machine learning (ML) have gained prominence in disease diagnosis, notably in the domain of medical image recognition [54]. By conducting a quantitative meta-analysis of prior studies, we have obtained the regional brain activity alterations relatively consistently involved in patients with MHE, thereby establishing a foundation for demarcating specific brain regions via AL and ML. Moreover, using AI and ML for the comprehensive analysis of rs-fMRI data could enhance the precision of MHE diagnosis, warranting further investigation in future research.

This study had some limitations. First, we included studies using different resting-state modes. Subgroup meta-analyses exploring the analytical approach exhibited consistency with the main analysis. However, it should be noted that their divergent theoretical foundations may have implications for the meta-analysis. Second, there might be potential heterogeneity in the demographic parameters of MRI scanners and image-processing procedures. Given the lack of data, it was not possible to carry out subgroup meta-analyses, such as magnetic field strength, smoothing, smooth kernel, and head motion parameters. Finally, because all studies included in this meta-analysis were from China, its universality across different ethnicities or demographics may be limited. Future studies should investigate the multiethnicity of the brain regions detected in this meta-analysis.

## 5. Conclusions

In summary, this was a quantitative meta-analysis utilizing the most recent size-signed differential mapping approach, with a TFCE-corrected threshold, to investigate spontaneous brain activity alterations in patients with MHE. Our findings indicated that MHE patients demonstrated decreased spontaneous brain activity in the left superior frontal gyrus and median cingulate/paracingulate gyri of the brain, possibly implicated primarily in spatial working memory and emotional disorders. These findings may provide useful insights into the underlying neural mechanisms of brain dysfunction in patients with MHE and guide further research.

## Figures and Tables

**Table 1:** Demographic and clinical characteristics of the studies included in the meta-analysis.

	Patient Information								Healthy Controls				
Study	Types	No	Female (%)	Mean Age (Year)	NCT-A (s)	DST (Score)	Ammonia (μmol/L)	Methods	No	Female (%)	Mean Age (Year)	NCT-A (s)	DST (Score)
Chen et al., 2012 [10]	MHE	22	9.09	53.10 ± 7.70	NA	22.80 ± 6.10	NA	ALFF	19	15.79	51.30 ± 7.80	NA	43.60 ± 9.30
	NMHE	18	11.11	50.60 ± 8.80	NA	40.40 ± 11.10	NA	ALFF	19	15.79	51.30 ± 7.80	NA	43.60 ± 9.30
Chen et al., 2012 [24]	MHE	18	11.11	54.80 ± 6.10	NA	23.10 ± 4.40	NA	ReHo	18	11.11	51.60 ± 7.90	NA	43.70 ± 9.50
Ji et al., 2020 [25]	MHE	31	38.71	43.51 ± 7.24	70.60 ± 34.9	21.10 ± 4.10	71.72 ± 8.50	ALFF	33	48.48	46.87 ± 7.24	20.65 ± 8.43	44.05 ± 11.00
	NMHE	28	46.43	45.32 ± 8.17	34.24 ± 5.15	44.86 ± 9.05	55.20 ± 6.53	ALFF	33	48.48	46.87 ± 7.24	20.65 ± 8.43	44.05 ± 11.00
Jiang et al., 2017 [26]	MHE	22	31.82	53.60 ± 1.50	NA	NA	NA	fALFF	13	38.46	53.80 ± 1.70	NA	NA
Ni et al., 2012 [11]	MHE	20	35.00	55.00 ± 7.00	72.80 ± 16.71	23.15 ± 8.17	69.06 ± 26.13	ReHo	25	48.00	55.00 ± 8.00	46.32 ± 9.09	44.68 ± 8.28
	NMHE	27	25.93	51.00 ± 6.00	45.78 ± 8.53	40.11 ± 8.80	51.10 ± 33.54	ReHo	25	48.00	55.00 ± 8.00	46.32 ± 9.09	44.68 ± 8.28
Qi et al., 2012 [12]	MHE	14	21.43	56.57 ± 9.19	NA	NA	38.58 ± 25.55	ALFF	17	29.41	54.35 ± 9.10	NA	NA
Shi et al., 2015 [27]	MHE	12	33.33	53.60 ± 9.40	86.92 ± 32.04	23.67 ± 7.08	20.83 ± 8.02	ALFF	12	41.67	53.60 ± 8.40	42.92 ± 11.38	41.33 ± 10.25
Shi et al., 2015 [28]	MHE	32	28.13	45.31 ± 8.96	117 ± 29.50	27.19 ± 5.13	NA	ReHo	34	29.41	46.62 ± 8.78	77.00 ± 17.00	44.26 ± 5.58
	NMHE	30	30.00	43.57 ± 10.24	85.00 ± 13.00	41.70 ± 4.85	NA	ReHo	34	29.41	46.62 ± 8.78	77.00 ± 17.00	44.26 ± 5.58
Sun et al., 2018 [13]	MHE	30	20.00	48.80 ± 12.20	NA	NA	NA	ReHo	64	28.13	46.80 ± 9.70	NA	NA
	NMHE	32	12.50	46.30 ± 9.20	NA	NA	NA	ReHo	64	28.13	46.80 ± 9.70	NA	NA
Wu et al., 2014 [29]	MHE	17	47.06	55.58 ± 10.41	NA	NA	NA	ReHo	17	47.06	55.11 ± 10.19	NA	NA
Yang et al., 2022 [30]	MHE	25	36.00	47.80 ± 9.60	70.30 ± 14.40	20.80 ± 5.00	NA	ReHo	30	37.00	44.90 ± 7.20	36.40 ± 8.80	46.00 ± 9.90
	NMHE	27	33.00	47.30 ± 9.50	38.10 ± 12.90	43.60 ± 4.80	NA	ReHo	30	37.00	44.90 ± 7.20	36.40 ± 8.80	46.00 ± 9.90
Zhong et al., 2016 [14]	MHE	14	50.00	54.57 ± 10.57	57.63 ± 30.96	23.29 ± 10.99	NA	ALFF/fALFF	14	50.00	50.86 ± 9.38	21.33 ± 3.80	48.86 ± 10.29
Zhou et al., 2014 [31]	NMHE	17	47.06	48.00 (37–70)	30.48 ± 5.44	42.59 ± 6.36	30.24 ± 8.94	ALFF	14	50.00	49.50 (36–68)	21.33 ± 3.80	48.86 ± 10.29

Abbreviations: ALFF, amplitude of low-frequency fluctuation; DST, digit symbol test; fALFF, fractional amplitude of low-frequency fluctuation; MHE, minimal hepatic encephalopathy; NA, not available; NCT-A, number connection test type A; NMHE, liver cirrhosis without MHE; ReHo, regional homogeneity.

**Table 2:** Resting-state brain abnormalities among patients with MHE or NMHE and HCs in the main meta-analysis.

	Maximum			Cluster		Heterogeneity		
Brain Regions	MNI Coordinates, x, y, z	SDM Value	*p*-Value	No. of Voxels	Breakdown (no. of Voxels)	Q (*p*-Value)	I^2^ (%)	Egger’s Test *p* Value
**MHE vs. HCs**								
*MHE* > *HCs*								
None								
*MHE* < *HCs*								
Left superior frontal gyrus	−2, 28, 40	−6.884	~0	1736	Left superior frontal gyrus, medial, BA 8, BA 9, BA 24, BA 32 (596); Left median cingulate/paracingulate gyri, BA 24, BA 32 (310)	4.400495 (0.728953)	4.494176	0.446
Left median cingulate/paracingulate gyri	0, −42, 36	−4.656	0.003000021	467	Left median cingulate/paracingulate gyri, BA 23 (119);Right precuneus (135)	7.236337 (0.523467)	7.436439	0.142
**NMHE vs. HCs**								
*NMHE* > *HCs*								
None								
*NMHE* < *HCs*								
Right rolandic operculum	52, −12, 10	−3.215	0.0006513	125	Right rolandic operculum, BA 48 (99); Right superior temporal gyrus, BA 48 (7)	1.675306 (0.41893)	0.653348	0.895
Left precentral gyrus	−58, −6, 30	−3.679	0.000117183	123	Left precentral gyrus, BA 3, BA 4 (47); Left postcentral gyrus, BA 3, BA 4, BA 43, BA 48 (76)	0 (0.524043)	6.151155	0.943
Right postcentral gyrus	58, −6, 30	−2.842	0.002242744	12	Right postcentral gyrus, BA 3, BA 4, BA 43 (12)	1.104866 (0.399276)	3.466042	0.958
**MHE vs. NMHE as compared to HCs**								
*MHE* > *NMHE*								
None								
*MHE* < *NMHE*								
Left superior frontal gyrus	0, 30, 42	−3.821	0.000999987	1935	Left superior frontal gyrus, medial, BA 8, BA 9, BA 24, BA 32 (657); Left median cingulate/paracingulate gyri, BA 24, BA 32 (88)	8.954032 (0.667304)	2.479716	0.623
Left median cingulate/paracingulate gyri	0, −42, 36	−2.084	0.04400003	63	Left median cingulate/paracingulate gyri, BA 23 (40); Right median cingulate/paracingulate gyri, BA 23 (20)	16.213957 (0.429249)	23.814262	0.804
Right precuneus	4, −54, 44	−2.134	0.046999991	24	Right precuneus (24)	15.727232 (0.418632)	13.443153	0.786

Abbreviations: HCs, healthy controls; MHE, minimal hepatic encephalopathy; MNI, Montreal Neurological Institute; NMHE, cirrhotic patients without MHE; SDM, seed-based d mapping z score.

## Data Availability

Data are available on request due to restrictions of privacy or ethics; the data presented in this study are available on request from the corresponding author.

## Supplementary Materials

The following supporting information can be downloaded at: https://www.mdpi.com/article/10.3390/brainsci13060960/s1, Table S1: Search strategy in PubMed database. Table S2: Quality assessment checklist. Table S3: Quality assessment details. Table S4: Technique details of resting-state functional magnetic resonance imaging studies included in the meta-analysis. Table S5: Subgroup meta-analysis results in studies using functional magnetic resonance imaging method (amplitude of low-frequency fluctuations) showing minimal hepatic encephalopathy and healthy controls of brain regions. Table S6: Subgroup meta-analysis results in studies using functional magnetic resonance imaging methods (regional homogeneity) showing minimal hepatic encephalopathy and healthy controls of brain regions.
