# Supplementary material for: Altered Spontaneous Brain Activity in Cirrhotic Patients with Minimal Hepatic Encephalopathy: A Meta-Analysis of Resting-State Functional Imaging
Source: Brain Sci. 2023 Jun 16;13(6):960. doi: 10.3390/brainsci13060960 (PMC10296644; doi:10.3390/brainsci13060960)
Supplement: Supplementary file 1 [file brainsci-13-00960-s001.zip › brainsci-2436289-supplementary.pdf]

Supplementary Materials

Table S1. Search strategy in PubMed database

Table S2. Quality assessment checklist.

Table S3. Quality assessment details.

Table S4. Technique details of resting-state functional magnetic resonance imaging studies included in the meta-analysis.

Table S5. Subgroup meta-analysis results in studies using functional magnetic resonance imaging method (amplitude of low-frequency fluctuations) showing minimal hepatic encephalopathy and healthy controls of brain regions.

Table S6. Subgroup meta-analysis results in studies using functional magnetic resonance imaging methods (regional homogeneity) showing minimal hepatic encephalopathy and healthy controls of brain regions.

Table S1. Search strategy in PubMed database

Search number	Search Details
1	cirrhosis[Title/Abstract] OR cirrhotic[Title/Abstract] OR liver cirrhosis[Title/Abstract] OR hepatic fibrosis[Title/Abstract] OR hepatic encephalopathy[Title/Abstract] OR HE[Title/Abstract] OR minimal hepatic encephalopathy[Title/Abstract] OR MHE[Title/Abstract]
2	functional magnetic resonance imaging[Title/Abstract] OR functional MRI[Title/Abstract] OR fMRI[Title/Abstract] OR amplitude of low-frequency fluctuation[Title/Abstract] OR ALFF[Title/Abstract] OR fALFF[Title/Abstract] OR regional homogeneity[Title/Abstract] OR ReHo[Title/Abstract]
3	1 AND 2

Table S2. Quality assessment checklist (when criteria were partially met, 0.5 points assigned).

**Category 1: Participants**<br>**Score (0/0.5/1)**
1. Patients were evaluated prospectively, specific diagnostic criteria were applied, and demographic data were reported.
2. Comparison participants were evaluated prospectively psychiatric and medical illnesses were excluded.
3. Important variables (e.g., age, sex, illness duration, onset, medication status, handedness) were checked either by stratification or statistically.
4. Sample size per group > 10.
**Category 2: Methods for image acquisition and analysis**
5. Whole brain analysis was automated with no *a priori* regional selection.
6. Coordinates reported in a standard space.
7. The imaging technique used was clearly described so that it could be reproduced.
8. Measurements were clearly described so that they could be reproduced.
**Category 3: Results and conclusions**
9. Statistical parameters for significant and important non-significant differences were provided.
10. Conclusions were consistent with the results obtained and the limitations were discussed.
TOTAL /10

Table S3. Quality assessment scores of included studies.

Studies	1	2	3	4	5	6	7	8	9	10	Total
Chen et al. 2012 [10]	1	1	1	1	1	1	1	1	1	1	10
Chen et al. 2012 [24]	1	0.5	1	1	1	1	1	1	1	1	9.5
Ji et al. 2020 [25]	1	1	1	1	1	1	1	1	1	1	10
Jiang et al. 2017 [26	1	0.5	1	1	1	1	1	1	1	0.5	9
Ni et al. 2012 [11]	1	1	1	1	1	1	1	1	1	1	10
Qi et al. 2012 [12]	1	1	1	1	1	1	1	1	1	1	10
Shi et al. 2015 [27]	1	1	1	1	1	1	1	1	1	0.5	9.5
Shi et al. 2015 [28]	1	0.5	1	1	1	1	1	1	1	0.5	9
Sun et al. 2018 [13]	1	1	1	1	1	1	1	1	1	1	10
Wu et al. 2014 [29]	1	1	1	1	1	1	1	1	1	0.5	9.5
Yang et al. 2022 [30]	1	1	1	1	1	1	1	1	1	1	10
Zhong et al. 2016 [14]	1	1	1	1	1	1	1	1	1	1	10
Zhou et al. 2014 [31]	1	1	1	1	1	1	1	1	1	0.5	9.5

Table S4. Technique details of resting-state fMRI studies included in the meta-analysis

Study	MR scanner	Software	Smoothing kernel (mm)	Statistical threshold	Coordinate system	Normalization technique	Temporal filtering (Hz)	Level of head motion
Chen et al. 2012 [10]	1.5 T TOSHIBA	DPARSF, REST	4	$p < 0.005$, AlphaSim corrected	MNI	spatial normalization into MNI	0.01–0.08	2.0 mm translation or 2.0° rotation
Chen et al. 2012 [24]	1.5 T TOSHIBA	REST	4	$p < 0.01$, AlphaSim corrected	MNI	spatial normalization into EPI	0.01–0.08	2.0 mm translation or 2.0° rotation
Ji et al. 2020 [25]	3.0 T GE	DPARSF	NA	$p < 0.05$, AlphaSim corrected	MNI	spatial normalization into MNI	0.01–0.08	NA
Jiang et al. 2017 [26]	3.0 T GE	REST	4	$p < 0.05$, corrected	MNI	spatial normalization into MNI	0.01–0.08	NA
Ni et al. 2012 [11]	3.0 T Siemens	SPM8, REST	4	$p < 0.01$, FDR corrected	MNI	spatial normalization into MNI	0.01–0.08	1.0 mm translation or 1.0° rotation
Qi et al. 2012 [12]	1.5 T GE	SPM8, REST	8	$p < 0.05$, AlphaSim corrected	MNI	spatial normalization into MNI	0.01–0.08	1.0 mm translation or 1.0° rotation
Shi et al. 2015 [27]	3.0 T Phillips	DPARSF	4	$p < 0.05$, AlphaSim corrected	MNI	spatial normalization into EPI	0.01–0.08	2.0 mm translation or 2.0° rotation

Shi et al. 2015 [28]	3.0 T Siemens	DPARSF	4	$p < 0.05$, AlphaSim corrected	MNI	spatial normalization into MNI	0.01–0.08	1.5 mm translation or 1.5° rotation
Sun et al. 2018 [13]	3.0 T GE	DPABI	4	$p < 0.05$, FDR corrected	MNI	spatial normalization into MNI	0.01–0.08	2.0 mm translation or 2.0° rotation
Wu et al. 2014 [29]	3.0 T Phillips	DPARSF	4	$p < 0.005$, AlphaSim corrected	MNI	NA	0.01–0.08	1.0 mm translation or 1.0° rotation
Yang et al. 2022 [30]	3.0 T GE	SPM8, DPARSF	NA	$p < 0.05$, AlphaSim corrected	MNI	spatial normalization into MNI	0.01–0.08	2.0 mm translation or 2.0° rotation
Zhong et al. 2016 [14]	3.0 T Phillips	DPARSF, REST	4	$p < 0.05$, AlphaSim corrected	MNI	spatial normalization into MNI	0.01–0.08	1.0 mm translation or 1.0° rotation
Zhou et al. 2014 [31]	3.0 T Phillips	DPARSF	4	$p < 0.05$, AlphaSim corrected	MNI	NA	0.01–0.08	NA

Abbreviations: DPABI, Data Processing and Analysis of Brain Imaging Toolbox; DPARSF, Data Processing Assistant for resting-state Functional Magnetic resonance Imaging; FDR, false discovery rate; fMRI, functional magnetic resonance imaging; MNI, Montreal Neurological Institute; NA, not available; REST, Resting State Functional Magnetic Resonance Imaging Data Analysis Toolkit; SPM, Statistical Parametric Mapping software.

Table S5. Subgroup meta-analysis results in studies using fMRI method (ALFF) showing MHE vs HCs of brain regions.

Brain region	MNI coordinates X Y Z	SDM Z score	P	No. of voxels	Cluster breakdown (no. of voxels)
MHE patients > HCs					
None					
MHE patients < HCs					
Left superior frontal gyrus	2,34,44	-4.791	0.000999987	697	Left superior frontal gyrus, medial, BA 8, BA 9, BA 24, BA 32 (408)
Right median cingulate / paracingulate gyri	2,-42,36	-4.289	0.000999987	809	Left median cingulate / paracingulate gyri, BA 23 (206)<br>Right median cingulate / paracingulate gyri, BA 23 (217)<br>Right precuneus, BA 5, BA 23 (146)

Abbreviations: ALFF, amplitude of low-frequency fluctuations; BA, Brodmann area; fMRI, functional magnetic resonance imaging; HCs, Healthy controls; MHE, minimal hepatic encephalopathy; MMNI, Montreal Neurological Institute; SDM, signed differential mapping; SDM-Z, Seed-based d Mapping Z score.
Cluster extent threshold: 10 voxels.

Table S6. Subgroup meta-analysis results in studies using fMRI method (ReHo) showing MHE vs HCs of brain regions.

Brain region	MNI coordinates X Y Z	SDM Z score	P	No. of voxels	Cluster breakdown (no. of voxels)
MHE patients > HCs					
None					
MHE patients < HCs					
Left median cingulate / paracingulate gyri	-2,26,32	-5.256	0.000999987	868	Left superior frontal gyrus, medial, BA 8, BA 9, BA 24, BA 32 (176)<br>Left median cingulate / paracingulate gyri, BA 24, BA 32 (145)<br>Right median cingulate / paracingulate gyri, BA 24, BA 32 (207)

Abbreviations: BA, Brodmann area; fMRI, functional magnetic resonance imaging; HCs, Healthy controls; MHE, minimal hepatic encephalopathy; MMNI, Montreal Neurological Institute; ReHo, regional homogeneity; SDM, signed differential mapping; SDM-Z, Seed-based d Mapping Z score.
Cluster extent threshold: 10 voxels.
